# Student Perspectives on Integrating an Oral Health Helpline Service Into Dental and Dental Hygiene Education

**DOI:** 10.1111/eje.13125

**Published:** 2025-06-13

**Authors:** Vanessa Johnson, Mario Brondani, HsingChi von Bergmann, Susan Grossman, Bruce Wallace, Leeann Donnelly

**Affiliations:** ^1^ Faculty of Dentistry University of British Columbia Vancouver British Columbia Canada; ^2^ Centre for Community Engaged Learning University of British Columbia Vancouver British Columbia Canada; ^3^ School of Social Work University of Victoria Victoria British Columbia Canada

**Keywords:** community health services, curriculum, dental education, dental hygiene education

## Abstract

**Introduction:**

The University of British Columbia's Faculty of Dentistry developed a helpline service to provide free oral health information, referrals to oral healthcare clinics and aid navigating public dental benefits. Over two academic years, 43 fourth‐year dental hygiene students and 29 dental students received education on being a virtual provider for the helpline and participated in experiential rotations as virtual providers. This qualitative study explored undergraduate dental and dental hygiene students' perspectives on integrating an oral health helpline into their curriculum.

**Methods:**

This project focuses on students' perspectives as part of a larger study employing constructivist grounded theory to develop a conceptual model for integrating an oral health helpline into a dental and dental hygiene school. Semi‐structured individual interviews were conducted with 5 dental and 7 dental hygiene students, audio‐recorded, transcribed verbatim and coded using NVivo 12 software. The coding process included initial, focused and theoretical coding, supported by constant comparative analysis. In initial coding, transcripts were reviewed for familiarisation and codes were generated by applying codes to concepts. Focused coding involved selecting a set of codes that were most prevalent and important to the analysis to develop categories that represent groups of codes. Theoretical coding consisted of refining the final categories and connecting the categories to each other.

**Results:**

Three major themes emerged as follows: perceived usefulness, educational components and institutional context. The helpline education improved students' understanding of barriers to oral health care. Dental hygiene students gained confidence in phone communication and providing virtual services, while dental students improved their ability to address patient questions, navigate care and share community resources. Experiential learning was crucial for greater understanding, with students recommending in‐person sessions, small group or one‐on‐one formats, organised resources and experienced instructors. Despite scheduling challenges, the helpline education could be integrated into existing relevant curriculum and distributed across academic years.

**Conclusion:**

Incorporating helpline education into dental programmes enhanced students' understanding of oral healthcare barriers, communications skills and ability to answer patient questions and share resources. This study's insights on helpline curriculum integration offer guidance for other dental programmes considering similar helpline initiatives.

## 1 Introduction

The coronavirus disease 2019 (COVID‐19) global pandemic disrupted access to routine and preventive oral health services in many countries, particularly during lockdown periods [1]. Reduced access to these services during lockdowns may have exacerbated oral health disparities among underserved populations [2, 3, 4]. The COVID‐19 lockdowns led to new teledentistry implementations in several countries, primarily for triage, follow‐up and nonprocedural care to reduce in‐person visits and improve access for those facing barriers to in‐person care [5]. Before the COVID‐19 pandemic, teledentistry was primarily used for triaging patients and delivering care over long distances [6]. Teledentistry has the potential to improve access to some services such as remote screening, diagnosis, health education, referrals, consultation and treatment planning and increase reach to underserved populations and in areas with limited availability of dental providers [7, 8, 9, 10]. Teledentistry also reduces costs for both patients and providers by minimising travel, time off work and in‐clinic overhead expenses [9, 11, 12]. In addition, teledentistry supports patient education, facilitates specialist consultations and can complement routine dental visits by having some visits be accommodated virtually [13].

However, teledentistry in dental and dental hygiene education varies by country as there are a notably higher number of studies conducted in the United States compared to other countries [14]. Most studies on teledentistry education have focused on undergraduate programmes in the USA, with few studies reporting teledentistry educational programmes from other countries including Canada, leaving the extent of teledentistry integration in dental education outside the USA largely unknown [14]. The academic literature on telemedicine education is also very limited and unevenly distributed globally, with a lack of published evidence from Canada and countries in the Scandinavian region, Europe and Africa, where telehealth activities are substantive [15]. Teledentistry training at different educational levels has been recommended in various studies, though barriers to teledentistry incorporation into the undergraduate level include an already overcrowded curriculum, faculty shortages and lack of legislation [16]. Teledentistry applications vary across dental and dental hygiene schools (DDHS) and are limited in Canada with only 30% of dental programmes and 63% of dental hygiene schools teaching teledentistry [14]. Although experiential practice is recommended to enhance teledentistry education and supplement lecture‐based teaching [17, 18], hands‐on practice has been limited to some dental hygiene programmes for teaching teledentistry in Canada [14]. Providing dental students with formal training and technical assistance would enhance their acceptance of teledentistry adoption [19]. However, most studies apply teledentistry in clinical settings while there is limited literature on its application in community oral health promotion and education [20].

Many countries have introduced telephone triage and advice services allowing people to speak to a nurse or general practitioner to support the public with health concerns and provide guidance to people seeking medical care [21, 22, 23, 24, 25]; however, they rarely address oral health‐related queries. Throughout the COVID‐19 pandemic, telephone helpline and triage services for dental care were used to provide consultations and referrals to prioritise emergency resources when dental clinics were restricted to providing emergency oral health care [26, 27, 28, 29]. There is a lack of literature on oral health telephone services in Canada and its integration into dental and dental hygiene education. Therefore, this study aimed to explore the viewpoints of dental and dental hygiene students on the oral health helpline education they received and its incorporation into a DDHS.

## 2 Methods

This qualitative research is part of a larger study employing constructivist grounded theory [30] to explore the integration of an oral health helpline into a DDHS. This study specifically focuses on the students' perspectives. Ethics approval was obtained from the University of British Columbia Research Ethics Board (H22‐02499).

### 2.1 Context

During the COVID‐19 pandemic, community organisations serving underserved populations requested assistance helping members navigate the oral healthcare system and having a contact to answer their dental‐related questions [31]. In response, the University of British Columbia's Faculty of Dentistry developed an oral health helpline service called Dental Link (DL) to facilitate oral healthcare system navigation by providing free information on oral health topics and public dental benefits and referrals to lower barrier oral healthcare clinics such as reduced‐cost dental clinics in British Columbia and University of British Columbia's community‐based preventive clinics that offer free dental hygiene services [32]. The oral health helpline seeks to address a community need for oral health‐related information and care navigation, with most users seeking information on where to access lower barrier oral health care in the initial two years of operations [32]. Fourth‐year dental and dental hygiene undergraduate students at University of Briths Columbia were involved with the helpline service as part of a pilot project to expose students to the needs of community members facing challenges attending in‐person services, while deepening students' understanding of how to meet the needs of underserved populations. Educating DDHS graduates on the social determinants of health and barriers to care may help prepare the future workforce to address oral health inequities [33, 34]. Implementing DL in curricula as a form of community engagement may broaden the students' awareness of barriers to oral health care by interacting with underserved communities [35, 36], while also providing an opportunity for students to practice helping the community navigate the oral healthcare system. The community gains a helpline resource offering oral health information, guidance with public dental benefits and service navigation support to improve access to oral health information particularly for communities experiencing oral health inequities.

Within the courses Dentistry Practice Management and Dental Hygiene Clinical Practice, fourth‐year dental and dental hygiene students received two didactic sessions introducing teledentistry, its applications, its connection to public health concepts and considerations for different applications. The students were also introduced to the DL helpline and trained on navigating relevant resources, including public dental benefit plans and the reduced‐cost dental clinics website, as well as providing services for the helpline. In addition, students participated in one or two 2–3 h experiential rotations to practice being virtual providers for DL. During these experiential rotations, the instructor initially reviewed relevant resources and how to address past cases, then the students responded to DL calls, emails or case scenarios. Over the two academic years of 2022–2023 and 2023–2024, didactic training was provided to 59 dental students and 48 dental hygiene students. In addition, 29 dental students and 43 dental hygiene students participated in the experiential rotation. The experiential rotations were part of a community rotation for 31 dental and 43 dental hygiene students at a partnering community health centre where students attend to clients' oral health needs.

The helpline didactic education was delivered on campus, while the experiential education was conducted at the community health centre. This setting offers students the opportunity for interprofessional collaboration with social workers and other professionals to provide comprehensive support to helpline users. All education was delivered by one licensed dental hygienist who developed the Dental Link helpline and the education. Questions from helpline users beyond the dental hygienist's scope were forwarded to a licensed dentist. Both dental and dental hygiene students received instruction from the same instructor to ensure consistency in content delivery.

### 2.2 Data Collection

Select dental and dental hygiene students who completed both the didactic training and the DL experiential rotation were purposefully recruited to participate in interviews, specifically looking for diverse experiences including opportunities to respond to DL users, online or in‐person education delivery and different levels of student engagement in the rotation. Students who did not complete both the didactic training and the experiential rotation or declined participation were excluded from the study. Participation was voluntary, and not all eligible students agreed to participate. Written informed consent was obtained from each participant, and interviews were conducted either in‐person in a private room or online via Zoom. The semi‐structured interview guide consisted of open‐ended questions without predefined answer choices, allowing participants to share their experiences freely. The interview guide questions focused on exploring the students' perspectives on the DL helpline, its integration into the DDHS and the impact of the helpline education including what the students valued and disliked about the education and how it influenced their understanding of the social determinants of health. Basic demographic information such as age, gender, prior education and current programme was collected. The interview guide was refined iteratively during data collection in accordance with constructivist grounded theory principles. A full list of interview questions is provided in the Appendix A. Data collection continued until theoretical saturation was reached when responses became repetitive and no new relevant codes or insights emerged. Interviews were audio‐recorded, transcribed verbatim and coded using NVivo 12 software, and supplemented with field notes and personal memos to maintain an audit trail.

### 2.3 Data Analysis

Data were analysed using a constructivist grounded theory approach [30], incorporating initial, focused and theoretical coding. The process began with line‐by‐line initial coding, allowing concepts to emerge closely from participants' language. These codes were then refined into focused codes, which helped identify patterns across transcripts. Theoretical coding was used to explore relationships among categories and begin developing conceptual linkages. Constant comparative analysis was conducted to compared new data with existing codes to refine categories. This process informed subsequent recruitment, while the interview guide was modified as needed. As themes developed, they were interpreted through relevant theoretical lenses such as the Technology Acceptance Model [37] and the Unified Theory of Acceptance and Use of Technology model [38] to support conceptual clarity. The conceptual framing was applied after the core themes had emerged from the data. To minimise bias, three researchers (‘VJ’, ‘DL’, ‘SG’) individually coded one transcript, compared codes for consistency and reached consensus coding. One researcher (‘VJ’) conducted, transcribed and coded all the interviews. Reflexivity is an active process of reflecting and developing insight into one's work as a researcher to guide future research actions, and it promotes the quality of grounded theory methods [39]. Reflexivity was used to improve trustworthiness and achieve rigour. For member checking, participants were offered the opportunity to review their interview transcript and a summary of the interpretations to verify, correct or change anything.

### 2.4 Positionality

Positionality refers to a researcher's worldview and position, which can influence the research process [40]. Therefore, it is important to disclose the researchers' positions and engage in reflexivity to address their impact on qualitative research [41]. The investigator‐interviewer (‘VJ’) was the developer of DL and the helpline curriculum. Students who were graded by ‘VJ’ were not part of the study population. Throughout data collection, data analysis and reporting, reflexivity was actively employed by being conscious of potential biases, remaining open to negative perceptions of DL and the helpline education, and maintaining an audit trail through memos. As the instructor and designer of the helpline education, ‘VJ’ focused the interviews on exploring students' perspectives on DL's incorporation into the DDHS.

## 3 Results

Five of 28 invited dental students (18%) and 7 of 19 invited dental hygiene students (37%) agreed to participate in the study. A total of 12 interviews with 5 dental students and 7 dental hygiene students were conducted, and interviews lasted between 37 and 60 min. Three themes emerged from analysis of students interviews on incorporating the DL helpline into their education: perceived usefulness, educational components and institutional context. In the following result section, when students' quotes were used to instantiate findings, please note that ‘D' is assigned to dental students and ‘DH’ is assigned to dental hygiene students. The main themes and subthemes are summarised in Table 1.

**TABLE 1:** Main themes and subthemes.

Main themes	Subthemes
Perceived Usefulness	Enhanced understanding of barriers to oral health care and available resources to help individuals navigate the oral healthcare system Strengthened ability to answer questions and share resources among dental students Increased comfortability with phone communication among dental hygiene students Limited impact due to short duration of helpline education
Educational Components	Interactive lectures preferred for the didactic component Importance of experiential learning for greater information retention Recommendations for experiential rotations: ○ Include real‐time interactions with DL users ○ Conduct sessions in‐person, and in small‐groups or one‐on‐one ○ Review information before the practical component ○ Provide clear documents and resources ○ Have a knowledgeable supervising instructor for guidance
Institutional Context	Majority of students believe the school has a role in helping the community navigate access to oral health care Few students viewed facilitating community access as the government's responsibility, a shared effort between the school and government or individuals' responsibility Barriers to incorporating the helpline curriculum include heavy course loads and busy schedules Facilitators for integrating the helpline curriculum include allocating dedicated time, embedding it into relevant existing curriculum and distributing content across multiple academic years

### 3.1 Perceived Usefulness

Students reported that the helpline education enhanced their understanding of barriers to oral health care, and they learned to use various resources to assist individuals in navigating the oral healthcare system, including public dental benefit programmes, reduced‐cost dental clinics and public documents such as the provincial dental fee guide.

Through Dental Link it seems like we can get greater exposure to more of these social determinants of health because I find that as dental students, we're only seeing one specific type of population at University of British Columbia. … So I think this can help us get greater exposure to more of these social determinants of health and then in turn learn how to navigate that and to support these patients. (D1, female, 24 years old)

The DL helpline education was perceived as strengthening dental students' ability to answer patient questions and share resources. Students reported that the helpline education made them feel more knowledgeable when answering oral health questions.

Dental Link has been a very good resource for us in learning how to approach this type of scenario where the patients are coming to us needing help, asking for either information or care. Before today, I always felt like it can be very overwhelming. … And having a general sense of what the framework is in approaching these cases, I think is not only helpful in communicating with these patients over the phone through Dental Link, but also something that we can take away for our patients in our own care. (D1, female, 24 years old)

The dental hygiene students reported increased comfort communicating over the phone and greater confidence in providing DL's virtual services;

I feel a lot more confident in being able to answer phone calls and answer questions that may arise. (DH1, female, 23 years old)

Students also noted drawbacks in the helpline education, including challenges in commuting to rotations, limited opportunities to respond to DL calls or emails and the education being only a small part of their curriculum.

It's only two rotations at Dental Link a year … it feels like something [students] have to go to once or twice, and then they don't have to think about it anymore. (DH6, female, 22 years old)

To address these issues, students suggested scheduling rotations with other in‐person activities, increasing the number of experiential rotations to provide opportunities to respond to users, and integrating the education more prominently into the curriculum.

### 3.2 Educational Components

Students shared their insights on their helpline education, focusing on the didactic education delivery, experiential learning and the educational support that enriched their experience. Students discussed various methods of delivering didactic education including lectures, online modules and online education delivery. While lectures were most preferred, it was noted that an interactive student component should be included for increased student engagement:

There needs to be like a lecture beforehand. … I think online lectures already have an inherent barrier to them because students are less engaged. Ideally it's in‐person. (D5, male, 26 years old)

Case scenarios … make [the education] more applicable than just sitting and having information disseminated to you. So making a lecture more interactive than just the traditional sit and listen format. (DH2, female, 30 years old)

Students emphasised the importance of having experiential learning because that type of learning is more impactful with greater information retention when the students apply the information:

We're given a lot of information, but if you don't apply it, the information doesn't really stay with you as well. … We should actually have some experience where we see what it entails and have experience being a part of [DL], like what we did today, being one of the providers. (D3, female, 26 years old)

Students valued practicing with case scenarios and role‐playing exercises. Other aspects that students appreciated in experiential rotations included real‐time interactions with users, being held in person, small group or one‐on‐one formats and first reviewing resources followed by practical experience.

Either small group or one‐on‐one would be perfect. In that small group, there was three of us and [the instructor] but we did kind of get that one‐on‐one feel too. Just because each of us would have a call and then [the instructor] would kind of help us. (DH3, female, 22 years old)

Having clear documents and resources outlining procedures when addressing helpline users' questions during the experiential rotation was perceived as critical by the students.

Everything was very clear. … it was really concise. And so it was really easy to be like, this person they have this certain insurance, for example. … I think the document on the information was the most helpful to my learning. (DH3, female, 22 years old)

Another beneficial aspect for the students' experiential rotation was having a knowledgeable instructor present to facilitate helpline interactions and provide guidance.

I don't think many of us had much exposure to it at that point and how to navigate those channels ourselves. … I think having [an instructor] there or another individual present who knows how to navigate them is very beneficial. (D4, male, 28 years old)

### 3.3 Institutional Context

Understanding student perspectives on the helpline education within the context of the DDHS is crucial. This includes assessing whether the helpline's objective of improving the community's access to oral health resources and services aligns with DDHS's values and considering logistical factors for effective implementation. Most students believed the DDHS has a role in helping the community navigate access to oral health services and that this could be facilitated through the helpline. Dental students' reasoning was that the DDHS is a role model and the only dental school in that province. Whereas the dental hygiene students explained how they already have community engagement rotations in their curriculum and the helpline would be another addition to further improve the community's accessibility to oral health services, promote the programme's available services and enhance communication and coordination between the community programmes and community members.

Since we already have these community rotations and we are trying to help the community with their oral healthcare needs, this is just one more aspect that would make it more accessible and easier for the community to find out about our services. (DH5, female, 21 years old)

In contrast, a few students perceived helping the community navigate access to oral health resources and services as being the government's responsibility, a collaboration between the DDHS and government or even community individuals themselves.

There should be some sort of government involvement in trying to encourage people or at least help people navigate access to oral healthcare. I think that maybe they could get some influence and insight through University of British Columbia. But I don't think the onus should be on University of British Columbia alone. … I think, as well, how much of the onus is on patients in general and their ability to navigate these channels and access to oral healthcare. (D4, male, 28 years old)

The students suggested that the helpline education should be integrated into relevant existing curriculum: Community Engagement for dental hygiene students and Practice Management for dental students. It appears that the dental hygiene students perceive the helpline service as a navigational tool for improving access to oral health services and resources, whereas the dental students viewed the helpline as beneficial for improving their communication with patients. In contrast, the helpline was not viewed as another method to deliver oral health care. As one dental hygiene student explained:

I feel like [the helpline education] fits a little bit more in community than clinical care because clinical care in my head [is] more of the hands‐on treatment that you're providing to the patient versus in community, we learn a little bit more about providing access, giving resources. And I feel like this is like a big part of that in terms of resource and accessibility to getting the services, not the services itself. (DH5, female, 21 years old)

One dental student discussed:

I think it should be part of our communications program and maybe Practice Management programs. … I think we can have a few more sessions where we actually have more practice communicating with the patients or our partners just to get more familiarity and being more comfortable with directing patients. (D2, female, 25 years old)

When asked about incorporating helpline education into their curricula, the students frequently reported scheduling issues, given their existing heavy course loads and their schedules already being very busy.

Our schedules are already so packed and if you're adding on another thing to the load that's already large enough, then it can be very overwhelming and maybe take away from what Dental Link is and how beneficial it could be to the students. (D1, female, 24 years old)

To facilitate integration of the new education, students suggested allocating dedicated time in their schedules, spreading the education across academic years and replacing other less beneficial parts of their existing curriculum.

## 4 Discussion

Undergraduate dental and dental hygiene students were trained to provide virtual services for an oral health helpline. This study aimed to explore the students' viewpoints on the helpline education and its incorporation into a DDHS. As a form of community engagement, the helpline education enhanced students' understanding of barriers to oral health care by exposing them to underserved individuals. The education improved the dental hygiene students' confidence in communicating over the phone and in providing DL's virtual services, while the dental students reported an improved ability to answer patient questions on care navigation or information and share community resources. However, the current helpline education is very limited in its time allocated in the dental and dental hygiene programme, which restricts its overall impact on the students. Nonetheless, the students perceived the helpline education as valuable and believed that the helpline education should be integrated into dental and dental hygiene curricula.

Given the absence of other oral health helpline education programmes in DDHS, the study findings were compared to existing teledentistry education programmes. Students highlighted the importance of experiential learning for deeper understanding and improved information retention. This aligns with Menhadji et al.'s findings, which showed increased confidence and competence among dentists and dental students after conducting their first video consultation with a patient [17]. The participating students appreciated practicing simulation exercises when real‐time user interactions were not possible. Patel et al. also noted increased comfort levels among students in leading a teledentistry visit after teledentistry simulation exercises [18]. Similarly to our study, Chen et al. highlighted the need for experienced instructors with the ability to assist students in teledentistry education [42]. Key recommendations for experiential rotations included conducting it in‐person, using small group or one‐on‐one formats, having available organised documents and resources and having an experienced instructor present to provide guidance.

Students identified scheduling issues as a barrier to integrating helpline education into their curriculum due to existing heavy course loads and busy schedules. Similar concerns were echoed by Amin et al. regarding the adoption of teledentistry in dental programmes, as well as faculty shortages, costs of required technology, lack of insurance coverage for teledentistry procedures and legal uncertainties [16]. The participating students, however, suggested incorporating the helpline education into relevant existing curriculum and proposed scheduling adjustments, such as allocating time for the education, increasing the exposure for experiential opportunities and spreading it across academic years. According to the students, the drawbacks of the helpline education involved commuting difficulties, limited real‐time interactions with users and the education's limited presence in their curriculum. Solutions included scheduling rotations on days with other in‐person activities and increasing experiential rotations.

While most students believed the DDHS should support the community in navigating the oral healthcare system, a few students felt this responsibility lies with the government, a collaboration between the DDHS and government or individuals. These students may view dental providers as not solely responsible for addressing oral health inequities. Kwon et al. found that one of the five contributors to dental students perceiving themselves as being a dentist is being proficient at domain‐specific knowledge and skills [43]. Thus, for dental educators, it is critical to recognise that the professional status of dental providers requires them to address societal health concerns [44], including oral health inequities. One's sense of social responsibility is influenced by various factors including societal position, politics, professionalism, economics and philosophy [45, 46, 47]. Our findings indicate a need to shift students' mindsets from solely prioritising clinical competence to also instilling humanistic values to fulfil their social contract, as recommended by Davis et al. [48] However, this is only possible with the DDHS valuing community activities beyond the clinical aspects of the profession [49]. Through the helpline education, students can practice helping individuals navigate barriers to oral health care and understand public dental benefits.

For other DDHS considering the adoption of a similar helpline initiative, we recommend integrating helpline education into existing courses to ensure curricular alignment and minimise scheduling conflicts. Experiential components should include multiple rotations, ideally with real‐time or simulated interactions, and conducted in small groups or one‐on‐one formats. These sessions should be supported by knowledgeable instructors and accompanied by clear, well‐organised resource materials. Additionally, framing the helpline as a form of community engagement may reinforce students' sense of professional responsibility and social accountability.

## 5 Limitations

This study was conducted at a single institution and may have many other institutional specific variables that may not be present elsewhere. The purposive sampling approach of this study further restricts generalisability to the broader student population. While this study included a small sample size of 12 participants that may be considered a limitation, this is consistent with constructivist grounded theory methodology, which prioritises depth, diversity of perspectives and theoretical saturation over generalisability. Nonetheless, the findings may not capture the full range of student experiences, and future studies with larger or more varied samples could provide additional insights. Social desirability bias is another potential limitation as participants may have felt influenced to provide favourable responses rather than express their true opinions, especially as the interviewer was also the instructor and developer of DL, despite assurances that their academic standing would be unaffected.

## 6 Conclusion

Incorporating the helpline education into dental and dental hygiene programmes enhanced the students' understanding of barriers to oral health care, communications skills and ability to answer patient questions and share community resources. The short curricular time of the helpline education in the dental and dental hygiene programmes limited its impact on the students. The students perceived the helpline education as valuable and expressed support for its integration into dental and dental hygiene curricula. Experiential learning, through real‐time interactions and simulations, is essential alongside didactic content. Moving forward, this study's findings will inform modifications to the ongoing investigation into faculty perspectives on integrating the oral health helpline education into DDHS programmes. Additionally, a formal evaluation of the helpline education will be conducted. This study's insights into the development of the helpline curriculum could be used by other DDHS considering the adoption of a similar helpline.

## Conflicts of Interest

The authors declare no conflicts of interest.

## Data Availability

The data that support the findings of this study are available from the corresponding author upon reasonable request.

## Appendix A

### Interview Guide

Demographics.

Age: Gender:

Educational training: Programme:

Interview Guide Questions:

Objective 1: *To explore the viewpoints of students on an oral health helpline and its incorporation into dental educational institutions*.

In the beginning of the COVID‐19 pandemic, I interviewed our partnered community organisations and their clients on their needs, and they requested a contact for asking dental‐related questions. In response, an oral health helpline called Dental Link was developed to facilitate access to oral health information and care by providing general oral health information, referrals to suitable dental offices such as reduced‐cost dental clinics and help navigating public dental insurance programmes. The helpline is accessible through phone call and email and is available to any resident in British Columbia.

1. What are your thoughts on this oral health helpline service?
  - Possibly probing question: Benefits and drawbacks?
2. What are your thoughts on whether our dental educational institution should help the community in navigating access to oral health care?
3. What are your thoughts on incorporating the Dental Link helpline into dental and dental hygiene education?

Objective 2: To explore the impact of Dental Link education on undergraduate dental and dental hygiene students.

4 How has the Dental Link education influenced your understanding of the social determinants of health by engaging with the community?
5 Was there anything else you gained or did not like about the Dental Link education?
6 What would improve the Dental Link education?

Objective 3: *To explore the viewpoints of students on teledentistry and its incorporation into dental educational institutions*.

Teledentistry has been defined as ‘*the use of telehealth systems and methodologies in dentistry’* [50]. The uses of teledentistry can be divided into four categories: teleconsultation, telediagnosis, teletriage and telemonitoring [51].

7 What are your thoughts on teledentistry?
8 What are the benefits and drawbacks of teledentistry compared to in‐person care?
9 What are your thoughts on whether teledentistry should be incorporated into dental and dental hygiene education?
10 What do you think dental and dental hygiene students might gain from teledentistry education?
11 What should dental and dental hygiene students learn in teledentistry education?
12 What are the enablers and barriers in incorporating teledentistry into dental and dental hygiene education?

Concluding Questions (All participants)

- Who else might be an important person to interview to gain their insight into teledentistry and an oral health helpline in a dental educational institution?
- Would you be willing to partake in another interview in the future?

*All participants will be asked if they would like to review their personal transcript and/or a summary of the transcript interpretations to verify, correct or change anything.
